# Assessment of lytic therapy effect in patients with intermediate‐high risk pulmonary embolism for prevention of chronic thromboembolic pulmonary hypertension: A randomized, double‐blind trial

**DOI:** 10.1002/hsr2.2093

**Published:** 2024-06-11

**Authors:** Pejman Mansouri, Amir Mohsen Rashidi, Mohammad Hadi Mansouri, Masoumeh Sadeghi, Reihaneh Zavar, Afshin Amirpour, Seyedeh Melika Hashemi, Marzieh Taheri

**Affiliations:** ^1^ Hypertension Research Center, Cardiovascular Research Institute Isfahan University of Medical Sciences Isfahan Iran; ^2^ Cardiac Rehabilitation Research Center, Cardiovascular Research Institute Isfahan University of Medical Sciences Isfahan Iran; ^3^ Isfahan Cardiovascular Research Center, Cardiovascular Research Institute Isfahan University of Medical Sciences Isfahan Iran; ^4^ Tehran Heart Center Tehran University of Medical Sciences Tehran Iran

**Keywords:** anticoagulant therapy, CTEPH, pulmonary embolism, thrombolytic

## Abstract

**Background and Aims:**

This study aims to compare the effectiveness of thrombolytic therapy and anticoagulation in preventing chronic thromboembolic pulmonary hypertension (CTEPH).

**Method:**

A total of 60 patients with intermediate‐high risk pulmonary embolism (PE) were randomly assigned to receive either thrombolytic therapy (*n* = 30) or anticoagulation (*n* = 30).

**Results:**

Echocardiographic assessments demonstrated no significant differences between the two treatment approaches in terms of right ventricular size (RVS) (on discharge in thrombolytic group: 31.17 ± 3.43 vs. anticoagulant group: 32.73 ± 5.27, *p* = 0.912), tricuspid annular plane systolic excursion (TAPSE) (on discharge in thrombolytic group: 17.66 ± 2.39 vs. anticoagulant group: 16.73 ± 2.93, *p* = 0.290), and systolic pulmonary artery pressure (SPAP) (on discharge in thrombolytic group: 32.93 ± 9.73 vs. anticoagulant group: 34.46 ± 9.30, *p* = 0.840). However, significant changes were observed in all assessed parameters within each treatment group (*p* < 0.001). The 6‐month follow‐up showed no significant difference between the two groups in terms of CTEPH incidence (*p* = 0.781) or functional class of the patients (*p* = 0.135).

**Conclusion:**

Based on the findings of this study, neither thrombolytic therapy nor anticoagulation demonstrated superiority over the other in reducing adverse outcomes associated with intermediate‐high risk PE, including right ventricular size, SPAP, TAPSE, or CTEPH.

## 1 INTRODUCTION

Pulmonary embolism (PE) is a potentially fatal condition that may lead to early mortality in the acute phase; however, the survivors may face chronic adverse events. Residual thrombi and microscopic vasculopathy subsequent to acute PE episodes are recognized factors contributing to the development of a life‐threatening condition that is known as chronic thromboembolic pulmonary hypertension (CTEPH).^1^, ^2^

Although approximately a quarter of patients diagnosed with CTEPH have no confirmed history of PE, CTEPH is the most critical long‐term complication of PE that carries significant morbidity and mortality.^3^, ^4^ Previously, CTEPH was considered a rare post‐pulmonary event with an incidence rate of 0.1%–0.5% in PE survivors.^5^ However, recent investigations have revealed remarkable higher rates accounting for 0.4%–8.8% of the PE patients.^5^, ^6^, ^7^

Understanding the process of CTEPH development following an acute PE, incidence, and the associated risk factors provides an improved view for the early diagnosis and prophylaxis of the patients at increased risk. The most significant factors include idiopathic PE, the previous history of venous thromboembolism, more severe perfusion defect, and the higher elevation in systolic pulmonary artery pressure (SPAP) at the time of acute PE incidence.^7^, ^8^, ^9^, ^10^ The effect of the management of an acute PE and CTEPH has not been well‐documented.

Anticoagulation is the primary management for acute submassive PE that prevents future embolic events recurrence, mainly fatal PE with risk of early death. Nevertheless, pulmonary perfusion restoration can be achieved by thrombolytic therapy. Besides, this approach decreases the pressure and resistance of the pulmonary circulation and can cause right ventricular function improvement.^11^

According to the guidelines, thrombolytics are the first‐line treatment among hemodynamically unstable massive PE patients, while those with submassive PE without hypotension require close observation, and thrombolytic can be administered if they develop cardiopulmonary deterioration. Hence, up until now, there is no strict indication for thrombolytic treatment rather than heparin in submassive PE.^12^ Accordingly, the current study, for the first time, aims to evaluate the effect of thrombolytic therapy versus anticoagulation for the prevention of adverse PE‐related events, CTEPH in particular.

## 2 METHODS

### 2.1 Study population

This is a double‐blinded randomized clinical trial (RCT) study, includes those with Intermediate‐high risk PE admitted from April 2018 to January 2022.

The Ethics Committee approved the study protocol based on code number “IR.MUI.MED.REC.1399.281”. Moreover, it has been registered into the Iranian Registry of Clinical Trials (IRCT20190301042873N1). The study protocol was explained to the patients (their legal guardians), they were reassured regarding the confidentiality of the personal information and signed written consent.

The patients with confirmed Intermediate‐high risk PTE^13^ diagnosed by an expert cardiologist without a history of previous PTE were included in the study. presence of any past medical history (e.g., COPD, significant valvular heart disease), absolute contraindications to fibrinolytic therapy (such as active bleeding, history of intracranial hemorrhage [ICH] or ischemic stroke in the past 6 months), and severe systematic disorders (e.g., Sepsis) were defined as the unmet criteria. The patients who withdrew the medication or were obliged to cease/alter the treatment because of severe adverse effects and those whose international normalized ratio (INR) was less than the target therapeutic range in over 70% of the assessments were excluded.

### 2.2 Randomization and blinding

The study has been conducted on all the patients who met the criteria. The patients were randomly assigned into one of the groups using Random Allocation Software by which each patient was provided with a particular number allocated him/her to one of the groups. The patients and cardiologists assessing cardiac function were blinded to whether patients received Alteplase or only anticoagulants for PTE management.

The first group was primarily treated with 100 mg alteplase infusion over 2 h, considering a response to the treatment. The patients allocated to the anticoagulant group received a intravenous infusion mirroring the volume and visual characteristics of Alteplase (utilized as a placebo).

Standard anticoagulant therapy, defining as 80 IU/kg bolus dose of heparin followed by 18 IU/h infusions or enoxaparin 1 mg/kg BD was administered for the second group.

Both groups initiated bridging therapy within 2 days after PTE incidence with oral anticoagulants (warfarin 5 mg), and intravenous treatment was ceased by achieving therapeutic levels of INR. Afterward, oral anticoagulation was continued for 6 months. INR level was assessed every 2–3 days, and then weekly for 2–3 weeks, until achievement of the desired range of 2–3. Afterward, INR was checked monthly.

### 2.3 Outcomes

The primary outcome of this study was to compare the effect of each treatment to prevent CTEPH incidence. Accordingly, the first step in the management of the patients before the medications was to perform echocardiography to assess the pulmonary pressure at study initiation. Other echocardiographs were performed at discharge and by 6 months after PTE incidence. All the echocardiographs were done by an expert echocardiography subspecialist, and CTEPH diagnosis was confirm by V/Q pulmonary scan. Secondary endpoints include right ventricular size, tricuspid annular plane systolic excursion (TAPSE), and SPAP, which were the assessed indices by echocardiography.

Besides, the patients’ age, gender, risk factor for PTE incidence, oral anticoagulant medication, intervention associated complications, length of hospital stays, and inferior vena cava (IVC) filter implementation were entered into the study checklist. They were also asked about their capability to perform daily chores and categorized according to the New York Heart Association classification. Bleeding was considered as major if it was ICH, required transfusions or intervention for hemodynamic deterioration. Other bleeding episodes considered as minor bleeding (e.g., hematuria, epistaxis).

### 2.4 Statistical analysis

The obtained data were entered into the Statistical Package for Social Sciences (SPSS Inc.) version 24. Descriptive data were presented in mean, standard deviation, percentages, and absolute numbers. To compare the qualitative data, chi‐square test and the quantitative data, an independent *t*‐test (or Mann–Whitney *U*‐test if the data distribution was non‐normal) was applied. Paired *t*‐test and a Wilcoxon test were administered. Logistic regression test was used to assess the chance of CTEPH incidence by each of the interventions. A *p*‐value of less than 0.05 was considered as a significant level.

## 3 RESULTS

**Figure 1:**
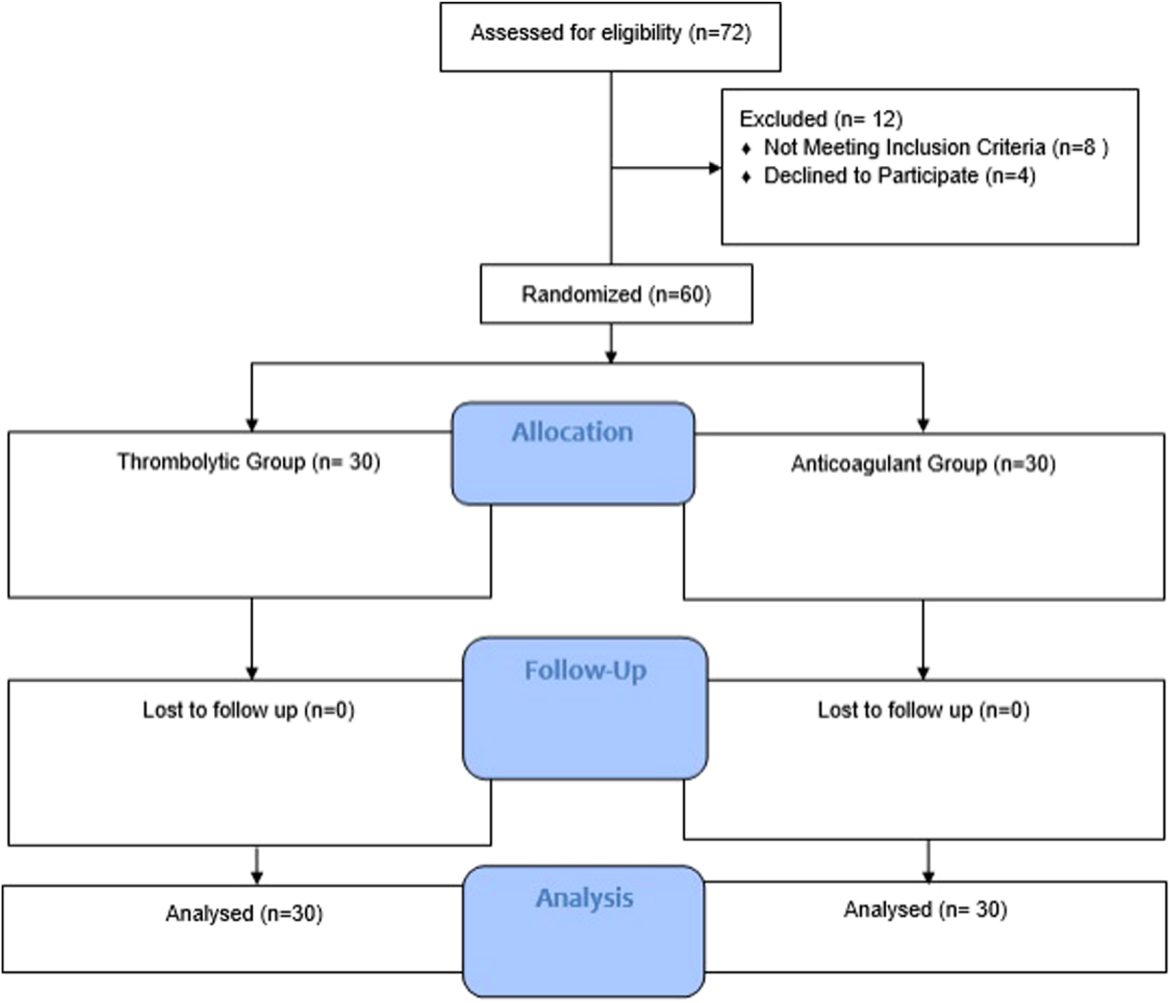
Demonstrates the CONSORT flow diagram of the study population.

Figure 1 demonstrates the CONSORT flow diagram of the study population. The current study has been conducted on 60 patients with Intermediate‐high risk PTE who were equally assigned into two groups of thrombolytic (*n* = 30) or anticoagulant (*n* = 30) therapy. The mean age ± standard deviation of the studied group was 58.5 ± 11.55 years which were predominantly consisted of males (*n* = 39, 65%). Both groups were similar in terms of age (*p*: 0.619) and gender distribution (*p* = 0.176). Besides, both were similar in terms of risk factors for PTE incidence and the administered oral anticoagulant medications (*p* > 0.05). Detailed information is demonstrated in Table 1.

**Table 1:** The demographic and medical characteristics of the studied population.

	Total (*n* = 60)	Thrombolytic therapy (*n* = 30)	Anticoagulant therapy (*n* = 30)	*p*‐value
Gender (male), *n* (%)	39 (65)	17 (56.7)	22 (73.3)	0.176
Age (years), mean ± standard deviation	60	57.8 ± 12.4	59.3 ± 10.8	0.619
Risk factors, *n* (%)				
Immobilization, *n* (%)	5 (8.33)	2 (6.7)	3 (10)	0.998
DM, *n* (%)	13 (21.6)	6 (20)	7 (23.3)	0.243
HTN, *n* (%)	15 (25)	8 (26.6)	7 (23.3)	0.493
Smoker, *n* (%)	13 (21.6)	6 (20)	7 (23.3)	0.243
BMI, mean ± standard deviation	26.3 ± 7.3	26.1 ± 6.4	25.9 ± 7.9	0.914
Serum creatinine, mean ± standard deviation mg·dL	0.9 ± 0.3	0.9 ± 0.4	1 ± 0.2	0.225
Deep venous thrombosis diagnosis, *n* (%)	10 (16.67)	6 (20)	4 (13.3)	0.488
Cancer, *n* (%)	2 (3.33)	0 (0)	2 (6.7)	0.492
Post operation, *n* (%)	10 (16.67)	6 (20)	4 (13.3)	0.488
Post partum, *n* (%)	2 (3.33)	0 (0)	2 (6.7)	0.492

Abbreviations: BMI, body mass index; DM, diabetes mellitus; HTN, hypertension.

Echocardiographic assessments revealed insignificant differences between the two therapeutic approaches regarding right ventricular size (*p* = 0.912), TAPSE (*p* = 0.290), and SPAP (*p* = 0.840). However, significant alterations were noted in all the parameters assessed in each of the groups (*p* < 0.001) (Table 2).

**Table 2:** The echocardiographic characteristics of the studied population.

Variable	Medication	Before intervention, mean ± standard deviation	On discharge, mean ± standard deviation	*p*‐value^a^	*p*‐value^b^ (the difference between the study initiation and on discharge)
RVS	Thrombolytic therapy (*n* = 30)	41.13 ± 3.78	31.17 ± 3.43	<0.001	0.912
	Anticoagulant therapy (*n* = 30)	42.57 ± 4.10	32.73 ± 5.27	<0.001	
*p*‐value^b^		0.165	0.178		
TAPSE	Thrombolytic therapy (*n* = 30)	12.18 ± 1.91	17.66 ± 2.39	<0.001	0.290
	Anticoagulant therapy (*n* = 30)	11.73 ± 2.40	16.73 ± 2.93	<0.001	
*p*‐value^b^		0.063	0.183		
SPAP	Thrombolytic therapy (*n* = 30)	54.16 ± 11.09	32.93 ± 9.73	<0.001	0.840
	Anticoagulant therapy (*n* = 30)	50.73 ± 15.92	34.46 ± 9.30	<0.001	
*p*‐value^b^		0.337	0.535		

Abbreviations: RVF, right ventricular function; SPAP, systolic pulmonary artery pressure; TAPSE, tricuspid annular plane systolic excursion.

^a^ Paired *t*‐test.

^b^ Independent *t*‐test.

Table 3 compares the severity of right ventricular enlargement and the severity of SPAP abnormality after applying each of the medications. According to this table, both the approaches led to significant improvement in the severity of RV enlargement (*p* < 0.001) and SAPS abnormality (*p* < 0.001); nevertheless, they did not statistically differ between the two treatment groups (*p* > 0.05).

**Table 3:** The comparison of echocardiographic dysfunctions severity in the studied groups.

Variable	Before intervention		On discharge		*p*‐value^a^ (anticoagulant therapy)	*p*‐value^a^ (thrombolytic therapy)
	Anticoagulant therapy (*n* = 30)	Thrombolytic therapy (*n* = 30)	Anticoagulant therapy (*n* = 30)	Thrombolytic therapy (*n* = 30)		
RV enlargement, *n* (%)						
No	0 (0)	0 (0)	9 (30)	9 (30)	<0.001	<0.001×
Mild	0 (0)	0 (0)	11 (36.7)	6 (20)		
Moderate	5 (16.7)	4 (13.3)	10 (33.3)	11 (36.7)		
Severe	25 (83.3)	426 (86.7)	0 (0)	4 (13.3)		
*p*‐value^b^	0.99		0.390			
Mean PAP abnormality						
No	0 (0)	0 (0)	20 (66.7)	21 (70)	<0.001	<0.001
Mild	10 (33.4)	11 (36.7)	8 (26.7)	4 (13.3)		
Moderate	13 (43.3)	12 (40)	1 (3.3)	4 (13.3)		
Severe	7 (23.3)	7 (23.3)	1 (3.3)	1 (3.3)		
*p*‐value	0.95^b^		0.39^c^			

Abbreviations: PAP, pulmonary artery pressure; RV, right ventricular.

^a^ Wilcoxon test.

^b^ Fisher's exact test.

^c^ Chi‐square test.

The 6‐month follow‐up assessment of the studied patients revealed an insignificant difference between the two groups regarding CTEPH incidence (10 patients in anticoagulant therapy group, 9 patients in thrombolytic, *p* = 0.781) and the patients’ function class (*p* = 0.135) (Table 4).

**Table 4:** The follow‐up echocardiography of the studied population.

Variable	Anticoagulant therapy (*n* = 30)	Thrombolytic therapy (*n* = 30)	*p*‐value^a^
CTEPH incidence (yes), *n* (%)	10 (33.3)	9 (30)	0.781
Function class, *n* (%)			
0	10 (33.3)	4 (13.3)	0.135
1	15 (40)	22 (73.3)	
2	5 (16.7)	4 (13.3)	

Abbreviation: CTEPH, chronic thromboembolic pulmonary hypertension.

^a^ Chi‐square.

The comparison of intervention‐associated complications revealed an insignificant difference between the two groups (*p* = 0.103). The complications in the thrombolytic treated group included 5 minor bleeding (2 epistaxis, 1 cutaneous hematoma, 2 hematuria) and ICH (1 patient), while only one patient in the anticoagulant therapy group presented heparin‐induced thrombocytopenia. No occurrences of in‐hospital mortality or hemodynamic instability were noted within our patient. The length of hospital stay was higher among the patients treated with anticoagulant (*p* = 0.002), while those who were treated with alteplase required more IVC filter implementation (*p* = 0.0.037) (Table 5).

**Table 5:** The comparison of complications.

Variables	Anticoagulant therapy (*n* = 30)	Thrombolytic therapy (*n* = 30)	*p*‐value
Complications incidence, *n* (%)	1 (3.3)	6 (20)	0.103^a^
Length of hospital stay (days), mean ± standard deviation	7.54 ± 1.71	6.19 ± 1.20	0.002^b^
IVC filter implementation, *n* (%)	1 (3.3)	4 (13.3)	0.037^c^

Abbreviation: IVC, inferior vena cava.

^a^ Fisher's exact test.

^b^ Independent *t*‐test.

^c^ Fisher's exact test.

## 4 DISCUSSION

Previous studies in the literature have progressively demonstrated that thrombolytic agents are capable of dissolving the pulmonary emboli that in turn leads to the improvement of pulmonary perfusion and right ventricular function. Nevertheless, this approach is recommended for the management of massive PEs only.^14^, ^15^ However, the efficacy of these agents for Intermediate‐high risk PEs has not been well‐documented. On the other hand, the administration of these agents should outweigh the risks of thrombolytic use, bleeding in particular.^16^

The current study tried to assess the efficacy of thrombolytic therapy versus anticoagulant agents for the prevention of PE‐related adverse outcomes in both in‐hospital and short‐term follow‐up of 6‐month evaluations. Accordingly, we found that none of the administered therapeutic approaches was superior over the other one in terms of CTEPH incidence, right ventricular size, TAPSE, SPAP and the patients’ function class. However, the requirement for IVC filter implementation was higher among those who were treated with thrombolytic.

Most of the studies in the literature have mostly evaluated the short‐term in‐hospital effects of these treatments and presented that thrombolytic therapy was accompanied by better outcomes as thrombolysis could reduce mortality and PE recurrence rate compared with anticoagulants.^11^, ^17^, ^18^, ^19^ Thrombolytics are associated with an increased probability of nonmajor bleeding, but major bleedings have been increased insignificantly^20^; however, Chen and colleagues have presented a twofolds increase in the risk of major bleedings by thrombolytic agents use. Therefore, they ignited a hypothesis regarding the administration of these agents in low doses.^20^

In the present study all of the patients, regardless of the therapeutic approach had abnormal SPAP on their hospital admission, while significant improvement was notified on‐discharge in both groups. Nevertheless, the comparison of the groups revealed an insignificant difference. This finding is in contrast with the study conducted by Ahmed et al.^2^ in Egypt, in which those treated with thrombolytic agents presented an statistically significant decrease in their SPAP. Similarly, the other study by Sharifi and colleagues who evaluated anticoagulant therapy alone or in combination with alteplase for the management of Intermediate‐high risk PE, stated that SPAP measured 48 h after admission was remarkably lower among those who received thrombolytic agents.^17^ Furthermore, Fusillo et al.^21^ compared these two treatments in 72 patients (35 ones treated with heparin and the remained ones managed by thrombolysis) and declared significant early improvement of mean pulmonary artery pressure in the thrombolytic‐treated than in the heparin group.

Right ventricular dysfunction and TAPSE were the other two aspects assessed in our study. We observed significant improvement in both parameters by on‐discharge assessments, but no difference was notified between the administered approaches. In agreement with our findings, Becattini et al.^11^ presented significant rehabilitation in RV function as well as a reduction in right ventricular end‐diastolic diameter by 24 h after treatment initiation regardless of therapeutic approach. In the PEITHO trial, individuals with intermediate‐risk PE receiving standard anticoagulation experienced a 5.6% incidence of death or hemodynamic decompensation within the initial 7 days post‐randomization, constituting the primary efficacy outcome. Conversely, a weight‐based dose of the fibrinolytic agent tenecteplase administered as a single‐bolus injection led to a notably reduced risk of the primary outcome (2.6%). However, this fibrinolytic treatment correlated with a 2.0% rate of hemorrhagic stroke and a 6.3% rate of major extracranial hemorrhage. As a result, the study concluded that in normotensive patients with intermediate‐risk PE, a single intravenous bolus of tenecteplase effectively decreased the composite primary outcome of early death or hemodynamic decompensation. Nevertheless, this treatment was also linked to a significant escalation in the risk of intracranial and other major bleeding.^22^

The great fear from inadequate management of elevated SPAP following an acute PE is to develop CTEPH. This condition imposes negative effects on the quality of life due to debilitating symptoms affecting physical capability.^8^ Nevertheless, we observed no difference by the administration of thrombolysis versus anticoagulants, neither by the incidence of CTEPH or by the assessment of their function class within 6 months after Intermediate‐high risk PE. Konstantinides et al. assessed the effects of thrombolytic therapy, tenecteplase, versus placebo on long‐term PE adverse events, they reported CTEPH in 2.1% of the cases versus 3.2% in the placebo group with insignificant difference; however, 33% of patients on thrombolytic therapy presented with persistent reduction in their function class.^23^ The observed CTEPH incidence in our study was higher than reported in some existing literature. This variance could potentially be attributed to diverse regional, racial, and ethnic factors. We hypothesize that the earlier assessment at 6 months, in contrast to the more commonly cited 2‐year period, may contribute to this disparity. Furthermore, we concur that the immediate post‐anticoagulation phase could result in temporary perfusion defects on V/Q pulmonary scans, which might resolve with extended anticoagulation. Additionally, our findings align with studies that have indicated a higher incidence of CTEPH when diagnosed without right heart catheterization, offering further justification for our results.^22^

### 4.1 Limitation

Although we tried our best to enhance the quality of this study, several limitations exist. Our sample size was relatively small, which might affect the generalization of our findings. Subsequently, a more extensive sample size is imperative to effectively consolidate and conclusively expound upon the findings. We followed the patients for 6 months which might be relatively short for an accurate assessment of the effectiveness of our intervention or the probable occurrence of adverse events.

## 5 CONCLUSION

According to the findings of this study, none of the thrombolytic therapy or anticoagulants was superior over the other to reduce the adverse outcomes of Intermediate‐high risk PE, including right ventricular size, SPAP, TAPSE, or CTEPH. It is imperative to note that these conclusions are preliminary and further investigation on a larger scale is necessary to validate these findings. The conclusions drawn from the study are merely hypothesis‐generating.

## AUTHOR CONTRIBUTIONS

**Pejman Mansouri**: formal analysis; methodology; validation; visualization; writing—original draft; writing—review & editing. **Amir Mohsen Rashidi**: conceptualization; resources; supervision. **Mohammad Hadi Mansouri**: conceptualization; investigation; project administration; supervision; writing—review & editing. **Masoumeh Sadeghi**: conceptualization; methodology; resources. **Reihaneh Zavar**: formal analysis; validation. **Afshin Amirpour**: supervision. **Seyedeh Melika Hashemi**: visualization; writing—review & editing. **Marzieh Taheri**: data curation; formal analysis; Methodology.

## CONFLICT OF INTEREST STATEMENT

The authors declare no conflicts of interest.

## TRANSPARENCY STATEMENT

The lead author Amir Mohsen Rashidi affirms that this manuscript is an honest, accurate, and transparent account of the study being reported; that no important aspects of the study have been omitted; and that any discrepancies from the study as planned (and, if relevant, registered) have been explained.

## Data Availability

The data that support the findings of this study are available on request from the corresponding author. The data are not publicly available due to privacy or ethical restrictions.
